# Neural Network-Based Prediction of Residual Paravalvular Leak in Bicuspid Aortic Valve TAVI Using CT-Derived Anatomical Features

**DOI:** 10.3390/biomedicines14040946

**Published:** 2026-04-21

**Authors:** Yijun Yao, Weili Jiang, Xinyue Yang, Jianyong Wang, Ruisi Tang, Yuan Feng, Yiming Li, Mao Chen

**Affiliations:** 1 Department of Cardiology, West China Hospital, Sichuan University, Chengdu 610041, China; yijun_yao@foxmail.com (Y.Y.); yangxy200101@163.com (X.Y.); ruisi_tang_acad@foxmail.com (R.T.); 2 Cardiac Structure and Function Research Key Laboratory of Sichuan Province, West China Hospital, Sichuan University, Chengdu 610041, China; 3 School of Computing and Artificial Intelligence, Southwest Jiaotong University, Chengdu 611756, China; jiangweili@swjtu.edu.cn; 4 College of Computer Science, Sichuan University, Chengdu 610065, China; wjy@scu.edu.cn

**Keywords:** bicuspid aortic valve, transcatheter aortic valve implantation, residual paravalvular leak, deep learning, computed tomography

## Abstract

**Background/Objectives**: Transcatheter aortic valve implantation (TAVI) in patients with bicuspid aortic valve (BAV) remains associated with higher rates of residual paravalvular leak (PVL), which confers a two-fold increase in mortality. Despite procedural optimization including balloon post-dilatation, a subset of patients exhibit residual ≥moderate PVL. Pre-procedural identification of these patients could guide procedural planning. **Methods**: We retrospectively analyzed 402 BAV patients who underwent TAVI with self-expanding valves and balloon post-dilatation between January 2016 and June 2024. A multi-modal deep learning model (Model B) was developed, integrating a 3D ResNet encoder for computed tomography (CT) imaging features with a multilayer perceptron (MLP) for clinical variables, fused via a cross-attention mechanism. Its performance was compared against a conventional model (Model A) combining clinical variables with manually derived CT measurements. Both models were evaluated on identical test folds using 5-fold stratified cross-validation. **Results**: Of 402 patients, 36 (9.0%) had residual ≥moderate PVL, associated with significantly larger aortic root dimensions at all anatomical levels and greater aortic valve calcification volume (median 887.6 vs. 559.2 mm^3^; *p* = 0.004). Model A achieved a mean AUC of 0.694 (95% CI: 0.596–0.792). Model B achieved a mean AUC of 0.822 (95% CI: 0.680–0.964), with a specificity of 0.971, accuracy of 0.881, and PPV of 0.860, while sensitivity was 0.429, reflecting the limited number of outcome events in this cohort. **Conclusions**: A multi-modal deep learning model integrating expert-segmented CT imaging with clinical variables demonstrated significantly improved discrimination over the conventional approach in this internal cohort for predicting residual PVL in BAV-TAVI, supporting the integration of segmentation-guided deep learning into pre-procedural TAVI planning. However, given the modest number of outcome events, external validation is required to confirm the generalizability of these findings.

## 1. Introduction

Transcatheter aortic valve implantation (TAVI) has emerged as a first-line therapy for patients with severe aortic stenosis [1,2]. With the expanded use of TAVI in younger individuals, long-term survival has become a critical consideration [3,4], particularly in patients with severe bicuspid aortic valve (BAV) stenosis. Indeed, the expanding TAVI population has driven interest in extending indications to BAV patients.

BAV is often associated with complex anatomical features that pose technical challenges for TAVI [5]. Although current clinical data demonstrate acceptable safety for TAVI in BAV [6], these very anatomical features can impede complete valve sealing, resulting in greater paravalvular leak (PVL) post-TAVI [7]. This is clinically significant, as residual ≥moderate PVL is independently associated with a two-fold increased risk of all-cause mortality during follow-up extending primarily to 2–3 years, based on a large individual patient data meta-analysis including a mix of early- and newer-generation valves [8], and patients with persistent ≥moderate PVL after re-intervention had significantly higher 1-year mortality compared with those in whom regurgitation was successfully reduced [9]. Various procedural optimization strategies, including balloon post-dilatation, have been employed to mitigate PVL [10,11]; however, these measures do not uniformly eliminate regurgitation, and a clinically important subset of patients are left with residual ≥moderate PVL. Computed tomography (CT) is valuable for predicting and understanding the mechanisms of PVL through annular sizing and assessment of calcification [12], yet conventional CT analysis typically captures only a limited number of standard dimensions and semi-quantitative calcification scores, which may not fully reflect the complex three-dimensional morphological determinants of prosthesis–annulus interaction.

Recently, deep learning has achieved significant advances in medical image analysis, enabling automated quantification of aortic valve morphology and pre-procedural planning [13]. Importantly, detailed segmentation of the aortic root and calcification on pre-procedural CT can serve as structured anatomical input for deep learning models, enabling them to learn complex three-dimensional morphological patterns—including geometric features, calcification distribution, and spatial relationships—that are impractical to capture through conventional manual measurements alone. Such an approach may reveal previously unrecognized anatomical determinants of residual PVL.

Here, we developed a multi-modal deep learning framework that integrates expert-segmented aortic root and calcification maps from pre-procedural CT with clinical variables to predict residual ≥moderate PVL in BAV-TAVI. We aimed to identify key anatomical and calcification patterns associated with residual PVL despite procedural optimization. These findings may provide imaging-based evidence to guide procedural planning and device strategy during BAV-TAVI and support a personalized interventional strategy.

## 2. Materials and Methods

### 2.1. Study Design and Ethical Approval

This was a retrospective, single-center, observational cohort study conducted at West China Hospital, Sichuan University, China. The study was approved by the Ethics Committee of West China Hospital (2022-1003). All participants provided written informed consent.

### 2.2. Study Population

**Figure 1:**
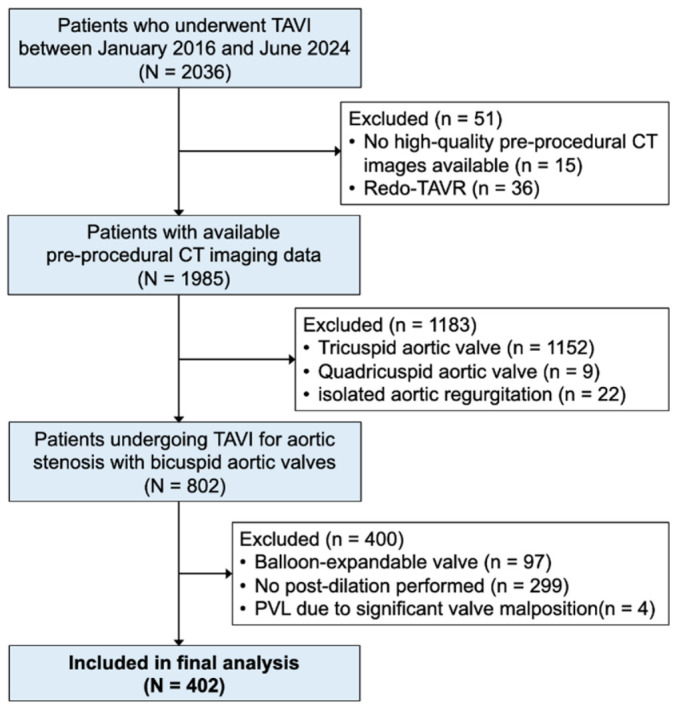
Study Flowchart.

A total of 2036 consecutive patients who underwent TAVI between January 2016 and June 2024 were screened for eligibility. After applying sequential exclusion criteria (Figure 1), 402 patients with BAV morphology confirmed on pre-procedural CT who received self-expanding transcatheter heart valves (THV) and underwent post-dilatation constituted the final analytic cohort. The exclusion criteria were as follows: (1) no high-quality pre-procedural CT images available (*n* = 15); (2) redo-TAVI (*n* = 36); (3) tricuspid aortic valve morphology (*n* = 1152); (4) quadricuspid aortic valve (*n* = 9); (5) pure aortic regurgitation (*n* = 22); (6) balloon-expandable valve implantation (*n* = 97); (7) no post-dilatation performed (*n* = 299); and (8) PVL due to significant valve malposition (*n* = 4).

### 2.3. Outcome Definition

The primary outcome was residual ≥moderate PVL, defined as ≥moderate PVL persisting after completion of all intra-procedural optimization measures (including balloon post-dilatation), assessed by intra-procedural transthoracic echocardiography. Patients were dichotomized into (1) the residual ≥moderate PVL group, regardless of subsequent management (second valve implantation, vascular plug closure, or conservative observation) and (2) the no-significant-residual-PVL group (<moderate PVL). The grouping was based on PVL severity at the conclusion of intra-procedural optimization, prior to any rescue interventions, to ensure the outcome reflected the anatomical determinants of incomplete valve sealing rather than the effect of secondary corrective procedures.

### 2.4. CT Image Acquisition and Expert Segmentation

#### 2.4.1. CT Image Acquisition

Patients underwent electrocardiogram-gated multidetector CT before TAVI. Approximately 80 mL of intravenous iodinated contrast was administered, with images reconstructed in the systolic phase (25–35% R–R intervals). CT data were analyzed using OsiriX (OsiriX Foundation, Geneva, Switzerland) by two experienced imaging physicians. For patients with bicuspid aortic valve morphology, valve sizing was guided by the supra-annular sizing algorithm when appropriate.

#### 2.4.2. Aortic Root and Calcification Segmentation

Aortic root and aortic valve calcification segmentation was performed on pre-procedural CT images by two experienced physicians, with verification by Y.F. (TAVI experience >10 years). The segmentation encompassed the complete aortic root anatomy from the left ventricular outflow tract to the ascending aorta. Calcification was segmented as a separate label, allowing independent quantification of its volume and relationship to the surrounding aortic root structures. All segmentations were performed using 3D Slicer v5.6.2 (Brigham and Women’s Hospital, Boston, MA, USA).

### 2.5. Multi-Modal Prediction Model (Model B)

#### 2.5.1. Overall Architecture

**Figure 2:**
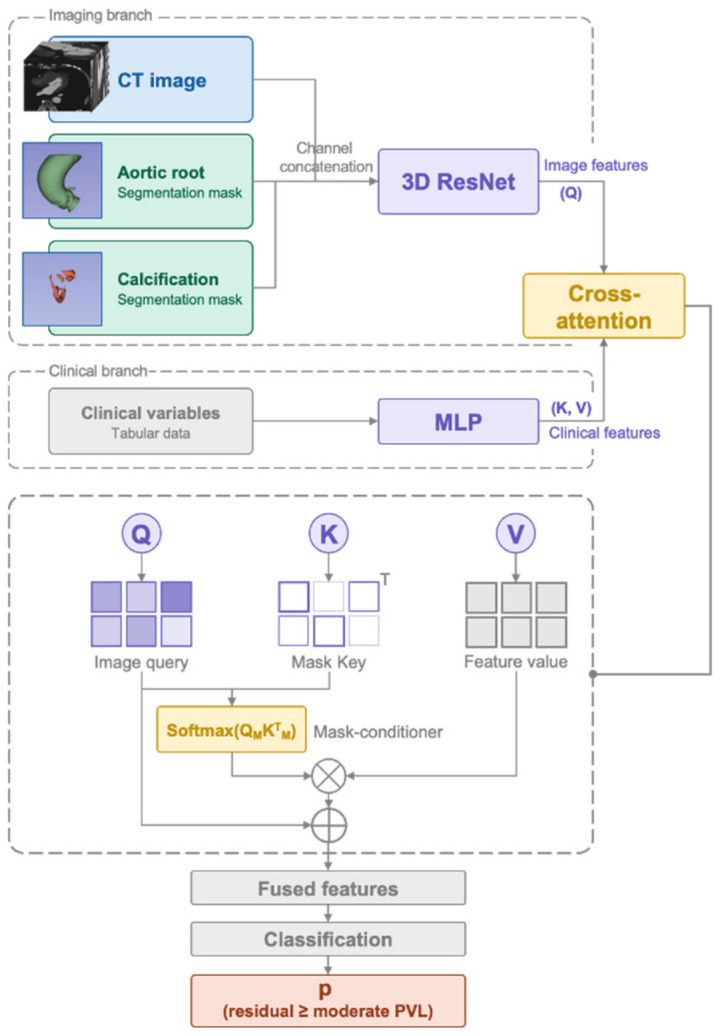
Architecture of the multi-modal deep learning model (Model B) for predicting residual paravalvular leak. *Q*, query; *K*, key; *V*, value (inputs to the cross-attention module); MLP, multilayer perceptron. Arrows indicate the direction of data flow.

A multi-modal deep learning framework was developed to predict residual ≥moderate PVL (Figure 2). The model comprised two parallel branches—an imaging branch and a clinical branch—whose learned representations were fused via a cross-attention mechanism before a final classification head.

#### 2.5.2. Imaging Branch: 3D ResNet Encoder

The imaging branch received multi-channel 3D volumetric input constructed from the pre-procedural CT. For each patient, three co-registered volumes were stacked along the channel dimension: (1) the original CT image, (2) the aortic root segmentation mask, and (3) the calcification region segmentation mask. This multi-channel representation allowed the model to jointly learn from raw image intensities and the spatial anatomical context provided by the expert segmentation. Volumetric features were extracted from the multi-channel input using a 3D ResNet-18 encoder operating on patches of 64 × 64 × 64 voxels. Details of image preprocessing and data augmentation are provided in Section 2.6.

#### 2.5.3. Clinical Branch: Multilayer Perceptron

The clinical branch received tabular clinical and procedural variables as input, including demographic characteristics, comorbidities, echocardiographic parameters, and procedural variables (as detailed in Section 2.6). Continuous variables were standardized (zero mean, unit variance), and categorical variables were encoded as binary indicators. The clinical variables were encoded by an MLP into a 64-dimensional feature embedding, which was subsequently used as the key (*K*) and value (*V*) inputs to the cross-attention fusion module described below.

#### 2.5.4. Cross-Attention Fusion and Classification

The feature representations from the imaging branch and the clinical branch were fused using a cross-attention mechanism. Specifically, the imaging features served as the query (*Q*), while the clinical features served as the *K* and *V*. The fused representation was computed as

$$F_{\mathrm{fusion}\;}=\mathrm{softmax}\left(\frac{QK^{T}}{\sqrt{d}}\right)V$$

where *d* denotes the dimension of the feature vectors. This design enabled the model to learn which imaging features were most relevant given each patient’s clinical context, effectively allowing the clinical profile to guide the spatial attention over anatomical regions.

The fused feature was then passed through a classification layer followed by a sigmoid activation function to output the predicted probability of residual ≥moderate PVL:

$$p=\sigma \left(WF_{\mathrm{fusion}\;}+b\right)$$

where *W* and *b* are the weight matrix and bias term of the classification layer, respectively, and *σ* denotes the sigmoid function.

#### 2.5.5. Training Strategy

The model was trained using a two-stage strategy designed to address the challenge of training a high-capacity 3D convolutional network on an event-sparse outcome (*n* = 36 events among 402 patients).

Stage 1 comprised proxy-task pretraining. Of the 402 patients, 192 were allocated to a pretraining subset. In this stage, the outcome label (residual ≥moderate PVL) was not used. Instead, the 3D ResNet encoder was trained to regress aortic valve calcification volume—a continuous, densely available anatomical measurement derived from expert segmentation that is an established mechanistic determinant of PVL. Calcification volume was computed as the total voxel volume of the expert-segmented calcification mask (mm^3^), log-transformed, and standardized to zero mean and unit variance across the pretraining subset. The encoder received the same three-channel volumetric input as in the downstream task (original CT, aortic root mask, calcification mask), followed by a global average pooling layer and a single linear regression head. Training used mean squared error loss, the Adam optimizer (initial learning rate 1 × 10^−4^, weight decay 1 × 10^−5^), batch size 4, for up to 100 epochs with early stopping on a 10% internal validation split of the pretraining subset. No PVL outcome information entered the model at any point during Stage 1.

Stage 2 comprised multi-modal fine-tuning and evaluation. The pretrained encoder weights were used to initialize the imaging branch of the full multi-modal model. The regression head from Stage 1 was discarded and replaced with the cross-attention fusion module and classification head described in Section 2.5.3 and Section 2.5.4. The model was then fine-tuned end-to-end on the remaining 210 patients using 5-fold stratified cross-validation (approximately 168 patients per fold for training and 42 for validation, with folds stratified by PVL outcome). All outcome-driven supervision occurred exclusively within Stage 2.

Because Stage 1 pretraining used only a continuous anatomical label (calcification volume) and did not use the PVL outcome, the allocation did not need to be stratified by outcome for unbiased pretraining. The 210-patient evaluation subset contained 35 patients with residual ≥moderate PVL (event rate 16.7%); the enrichment of outcome events in the evaluation subset was intentional, to preserve statistical power for cross-validated discrimination estimates in this event-sparse setting. As the pretraining stage did not receive outcome labels, this outcome-aware allocation does not constitute label leakage.

The model was optimized using binary cross-entropy loss:

$$L=-\frac{1}{N}\sum_{i=1}^{N}\left[y_{i}\log\left(p_{i}\right)+\left(1-y_{i}\right)\log\left(1-p_{i}\right)\right]$$

where *y_i_* denotes the ground-truth label for sample *i*, and *p_i_* denoted the predicted probability for the sample *i*.

#### 2.5.6. Model Interpretability Analysis

To visualize the anatomical regions driving Model B’s predictions, Gradient-weighted Class Activation Mapping (Grad-CAM) was applied to the 3D ResNet encoder. Feature maps from the final convolutional layer were weighted by their gradient-based importance with respect to the predicted class, producing a three-dimensional heatmap that was subsequently overlaid on the original CT volume. The resulting maps highlight voxel regions that contributed most to the model’s classification of residual ≥moderate PVL risk for each individual patient.

### 2.6. Model Training and Validation

For Model B (Stage 2 fine-tuning; see Section 2.5.5), a learning-rate scheduler was applied with a decay factor of 0.35 and a patience of 10 epochs. The model was trained for up to 200 epochs, with early stopping implemented using a patience of 40 epochs. For image preprocessing and augmentation, all images were resampled to a voxel spacing of 1.0 × 1.0 × 2.0 mm, intensity-normalized to [0, 1] using a window of [−79, 304], cropped around the foreground with a margin of 30 voxels, and resized to 192 × 128 × 96. During training, random cropping with a patch size of 64 × 64 × 64 and random intensity shifting (offset = 0.40, probability = 0.50) were applied. Training was conducted on one NVIDIA RTX 4090 GPU (NVIDIA Corporation, Santa Clara, CA, USA).

Model A was evaluated on the identical 210-patient cohort and fold assignments used for Model B (Section 2.5.5). Input features comprised established clinical, anatomical, and procedural predictors of PVL in BAV-TAVI, selected a priori from the literature rather than through data-driven methods, and were standardized per fold (zero mean, unit variance). Five classifiers were compared: L1- and L2-regularized logistic regression (regularization strength optimized via cv.glmnet), MLP (single hidden layer, 32 units, L2 decay = 0.001, up to 500 epochs), Random Forest (200 trees, max 16 terminal nodes, min node size = 5), and gradient boosting machine (100 trees, interaction depth = 3, shrinkage = 0.05, bag fraction = 0.8). Hyperparameters for the tree-based and MLP classifiers were set to recommended defaults rather than tuned by grid search, given the low event count (*n* = 36). Class-balanced sample weights were applied across all classifiers. The best-performing classifier was selected by 5-stratified-fold cross-validated AUC.

### 2.7. Statistical Analysis

Continuous variables were expressed as medians (interquartile range [IQR]) and compared using the Wilcoxon rank-sum test. Categorical variables were expressed as counts (percentages) and compared using the Chi-square test or Fisher’s exact test, as appropriate. Model discrimination was assessed using AUC. Pairwise model comparisons were performed using the Delong test. Clinical utility was assessed by decision curve analysis (DCA). Model calibration was assessed using reliability (calibration) curves and the Brier score, computed across all out-of-fold predictions from the 5-fold cross-validation. Calibration curves were generated using uniform binning of predicted probabilities, plotting the observed event proportion against the mean predicted probability within each bin. All statistical tests were two-sided, with *p* < 0.05 considered statistically significant. Deep learning models were implemented in Python 3.10.16 (Python Software Foundation, Wilmington, DE, USA) using PyTorch 2.7.0 (Meta AI, Menlo Park, CA, USA); statistical analyses were performed using R v4.3.2 (R Foundation for Statistical Computing, Vienna, Austria).

## 3. Results

### 3.1. Study Population and Baseline Characteristics

Of 2036 consecutive TAVI patients screened, 402 BAV patients who received self-expanding THVs and underwent post-dilatation constituted the final analytic cohort (Figure 1). Among them, 36 (9.0%) had residual ≥moderate PVL (≥moderate PVL group) and 366 (91.0%) had <moderate PVL (<moderate PVL group).

Baseline clinical, anatomical, echocardiographic, and procedural characteristics are presented in Table 1. Compared with the <moderate PVL group, patients with ≥moderate PVL were older (median 74 vs. 72 years; *p* = 0.031) and more frequently male (75.0% vs. 54.9%; *p* = 0.032) and had a lower prevalence of diabetes (5.6% vs. 19.1%; *p* = 0.041). Other comorbidities and the STS score were comparable between groups.

Anatomically, the ≥moderate PVL group was characterized by significantly larger aortic root dimensions at all levels: annular perimeter and area (*p* = 0.003 and 0.010), sinus of Valsalva perimeter (*p* < 0.001), sinotubular junction diameter (*p* < 0.001), LVOT perimeter (*p* = 0.007), and maximum ascending aortic diameter (*p* < 0.001). Aortic valve calcification volume was substantially higher in the ≥moderate PVL group (median 887.6 vs. 559.2 mm^3^; *p* = 0.004). Echocardiographic hemodynamic severity was similar between groups, though LVEF tended to be lower (*p* = 0.057) and LVEDD was significantly larger (*p* = 0.002) in the ≥moderate PVL group. Procedurally, the post-dilatation balloon size was larger in the ≥moderate PVL group (median 22 vs. 20 mm; *p* = 0.008), while THV type, size, generation, and access route were comparable.

### 3.2. Prediction Model Performance

#### 3.2.1. Model A: Conventional Approach

Model A, combining clinical and procedural variables with manually measured CT measurements (35 features in total), achieved an AUC of 0.694 (95% CI: 0.596–0.792) using the Random Forest classifier, which was selected as the best-performing model among five candidates (LR–L1, LR–L2, MLP, RF, GBM; Supplementary Table S1). The overall accuracy was 0.781, sensitivity was 0.657, specificity was 0.806, PPV was 0.488, and NPV was 0.930. The low PPV indicates that although Model A detected the majority of patients with residual PVL, it did so at the cost of a high false-positive rate—approximately four out of five patients flagged as high-risk did not actually experience residual PVL.

#### 3.2.2. Model B: Multi-Modal Deep Learning

Model B, the multi-modal deep learning framework integrating 3D ResNet-encoded CT imaging with clinical variables via cross-attention, achieved substantially superior performance. Across 5-fold cross-validation, Model B attained a mean AUC of 0.822 ± 0.114 (95% CI: 0.680–0.964), accuracy of 0.881 ± 0.038 (95% CI: 0.834–0.928), sensitivity of 0.429 ± 0.202 (95% CI: 0.178–0.680), specificity of 0.971 ± 0.049 (95% CI: 0.910–1.000), PPV of 0.860 ± 0.219 (95% CI: 0.588–1.000), and NPV of 0.896 ± 0.030 (95% CI: 0.859–0.933) (Table 2). The performance metrics for each individual fold are detailed in Supplementary Table S2.

#### 3.2.3. Head-to-Head Comparison

**Figure 3:**
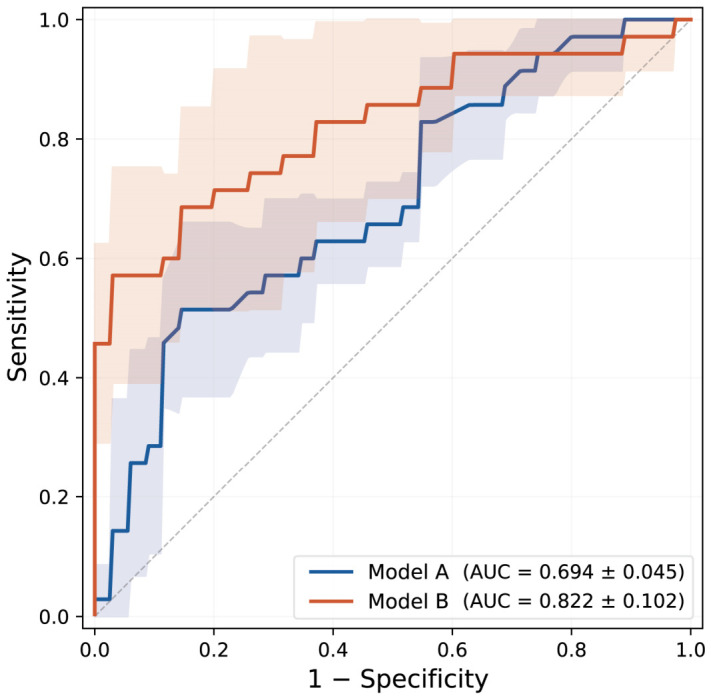
Receiver operating characteristic curves of Model A and Model B for predicting residual ≥moderate PVL after BAV-TAVI. The blue line represents Model A and the orange-red line represents Model B. The diagonal dashed line represents the reference line for a no-skill classifier (AUC = 0.5). The shaded bands around each ROC curve denote ±1 standard deviation across the 5 cross-validation folds.

Model B demonstrated significantly better discrimination than Model A (AUC: 0.822 vs. 0.694; Table 2). The most notable difference lay in specificity and PPV: Model B achieved a specificity of 0.971 vs. 0.806 for Model A, and a PPV of 0.860 vs. 0.488, indicating that a high-risk prediction from Model B was substantially more likely to be correct. This improvement came at the cost of lower sensitivity (0.429 vs. 0.657), reflecting a conservative decision boundary—Model B missed more true-positive cases but generated far fewer false alarms. Overall accuracy was higher for Model B (0.881 vs. 0.781). NPV was comparable between models (0.896 vs. 0.930), indicating that both reliably identified patients at low risk for residual PVL. The ROC curves for both models are presented in Figure 3.

#### 3.2.4. Model Calibration

**Figure 4:**
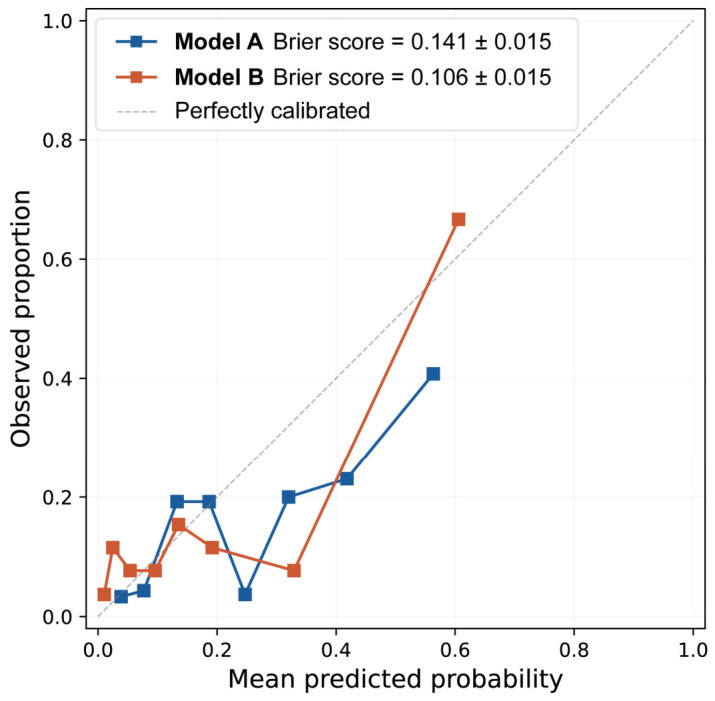
Calibration curves of Model A and Model B for predicting residual ≥moderate PVL after BAV-TAVI. Calibration was assessed on pooled out-of-fold predictions from 5-fold cross-validation. Model A (blue) yielded a Brier score of 0.141 ± 0.015, close to the no-information benchmark of 0.139 expected for a model predicting only the cohort event rate (16.7%). Model B (red) yielded a Brier score of 0.106 ± 0.015, indicating better-calibrated probability estimates. BAV, bicuspid aortic valve; PVL, paravalvular leak; TAVI, transcatheter aortic valve implantation.

Calibration was assessed on the pooled out-of-fold predictions (Figure 4). Model B achieved a Brier score of 0.106 ± 0.015, lower than Model A (0.141 ± 0.015) and below the no-information benchmark of 0.139 expected for a model predicting only the cohort event rate (16.7%), indicating that Model B carries probabilistic information beyond prevalence alone. The calibration curve of Model B approximated the diagonal at low predicted probabilities (0–0.2), where the majority of patients were classified, with slight under-prediction in the highest probability bin—a directionally favorable pattern for clinical use. The non-monotonic appearance of both curves should be interpreted in the context of the limited number of outcome events (*n* = 35), which yields sparsely populated probability bins.

#### 3.2.5. Grad-CAM Visualization Results

**Figure 5:**
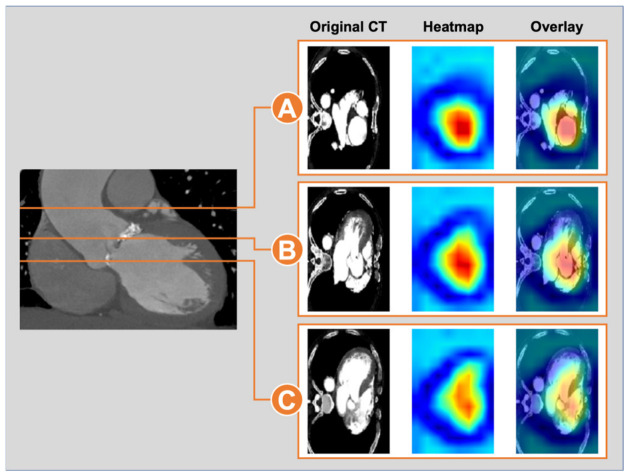
Grad-CAM for a representative bicuspid aortic valve patient with residual ≥moderate paravalvular leak predicted by Model B. Left panel: coronal reference view of the aortic root, with horizontal lines indicating the three axial levels displayed on the right. For each level, the original CT slice (left), the corresponding Grad-CAM heatmap derived from the final convolutional layer of the 3D ResNet encoder (middle), and the heatmap overlaid on the CT image (right) are shown. Warmer colors (red) denote voxels with greater contribution to the model’s prediction of residual ≥moderate PVL, whereas cooler colors (blue) denote minimal contribution. (**A**) Ascending aortic level: model attention is concentrated on the lumen and wall of the dilated ascending aorta. (**B**) Aortic annular level: attention localizes to the annulus and adjacent leaflet calcification, the principal anatomical interface for prosthesis sealing. (**C**) Left ventricular outflow tract (LVOT) level: attention extends into the LVOT and sub-annular region, where calcium protrusion and outflow geometry are known to compromise skirt apposition. Collectively, the spatially coherent activation across the ascending aorta–annulus–LVOT axis indicates that Model B’s prediction is driven by anatomically plausible, multi-level features of the aortic root rather than by isolated or off-target image regions.

Grad-CAM were generated for individual patients to visualize the anatomical regions driving Model B’s predictions (Figure 5). In a representative true-positive case, model attention was distributed coherently across three axial levels of the aortic root: the ascending aorta (Figure 5A), the aortic annulus and adjacent leaflet calcification (Figure 5B), and the LVOT (Figure 5C), suggesting that Model B’s discrimination is supported by anatomically plausible multi-level features rather than spurious image regions.

#### 3.2.6. Clinical Utility

**Figure 6:**
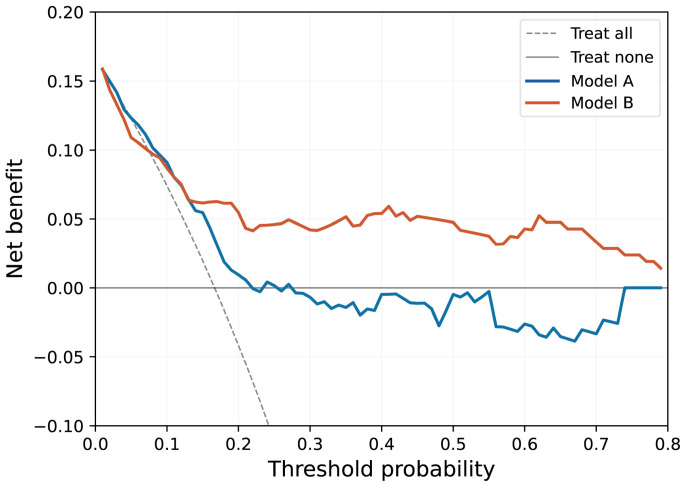
Decision curve analysis of Model A and Model B for predicting residual ≥moderate PVL after BAV-TAVI. Model A (blue line; conventional approach with Random Forest classifier) demonstrated a positive net benefit at threshold probabilities below approximately 0.27, falling below the “Treat None” line at higher thresholds. Model B (red line; multi-modal deep learning) maintained a positive net benefit across a broad range of threshold probabilities (0.01–0.80), consistently exceeding both reference strategies. At threshold probabilities above approximately 0.13, Model B provided a greater net benefit than Model A, with the advantage becoming increasingly pronounced at higher thresholds. The grey dashed line represents the “Treat All” strategy (intervene on all patients), and the black solid line represents the “Treat None” strategy (intervene on no patients; net benefit = 0). BAV, bicuspid aortic valve; PVL, paravalvular leak; TAVI, transcatheter aortic valve implantation.

Decision curve analysis demonstrated that Model B yielded a positive net benefit across a wide range of clinically relevant threshold probabilities (0.01–0.80), consistently outperforming Model A and both default strategies (Figure 6). In contrast, Model A provided net benefit only at very low thresholds, limiting its practical utility for clinical decision-making.

## 4. Discussion

### 4.1. Summary of Principal Findings

Residual ≥ moderate PVL after TAVI is associated with an approximately two-fold increase in all-cause mortality and higher rates of heart failure rehospitalization [8,9], yet remains difficult to predict pre-procedurally, particularly in patients with BAV whose complex anatomy predisposes them to incomplete valve sealing. In this work, we developed a multi-modal deep learning model (Model B) integrating expert-segmented CT imaging and clinical variables via a cross-attention mechanism to predict residual ≥moderate PVL in BAV-TAVI, and compared its performance against the conventional approach of combining clinical variables with manually derived CT measurements (Model A). Our major findings are as follows: (1) residual ≥moderate PVL occurred in 9.0% of BAV patients and was associated with significantly larger aortic root dimensions and greater calcification burden; (2) the conventional model combining clinical and manual CT parameters achieved an AUC of 0.694, confirming that anatomical information is essential for PVL prediction; (3) the multi-modal deep learning model achieved an AUC of 0.822 with a specificity of 0.971, representing a notable improvement over the conventional approach within the constraints of this retrospective dataset. Owing to the limited number of PVL events (*n* = 36), the performance estimates—particularly the high specificity and positive predictive value—should be interpreted with caution and may exhibit greater variability in larger or external samples. Collectively, these results suggest that segmentation-guided deep learning can extract richer anatomical information from pre-procedural CT than conventional measurements, meaningfully improving PVL prediction and supporting personalized decision-making during BAV-TAVI.

### 4.2. Anatomical Complexity in BAV Related to PVL

BAV, the most common congenital heart defect, presents unique anatomical challenges, including asymmetrical cusps, bulky calcification, and commissural fusion, which compromise symmetrical valve expansion and optimal sealing [14,15], resulting in lower device success [16,17]. Although the development of new-generation THVs has achieved more favorable results competitive with tricuspid valve TAVI [7,18], BAV-TAVI remains associated with higher rates of residual significant PVL, largely attributable to the adverse anatomical factors mentioned above. Larger dimensions of the aortic annulus and of the aortic root often necessitate larger THVs, hence increasing the risk of PVL [19]. The relationship between heavier aortic valve calcification and PVL severity has also been well established in prior studies [20,21]. In one study, the relationship between the localization of calcium in the aortic annulus and the localization of PVL was also highlighted [22]. These challenges may worsen when self-expandable valves are used. Unlike balloon-expandable THVs (BE THVs), self-expandable THVs (SE THVs), while better at conforming to the irregular BAV orifice, are less capable of achieving a fully circular configuration for optimal sealing, especially in earlier-generation THVs. Additionally, some SE THV platforms lack an external sealing skirt, further increasing the risk of paravalvular regurgitation. Notably, the preference for SE THVs in BAV patients with severe annular calcification—driven by concerns over annulus rupture with BE THVs—may inadvertently contribute to their higher observed PVL rates [18,23]. Indeed, a recent meta-analysis-based commentary highlighted the nuanced trade-offs inherent in this choice: while BEVs are associated with lower PVL and pacemaker implantation rates, they carry higher risks of annular rupture and elevated transvalvular gradients [24], underscoring the need for anatomy-driven, individualized device selection.

Our findings are consistent with this body of evidence. Patients with residual ≥moderate PVL demonstrated significantly larger aortic root dimensions at all anatomical levels—annulus, sinus of Valsalva, sinotubular junction, LVOT, and ascending aorta—and carried a substantially higher aortic valve calcification burden (median 887.6 vs. 559.2 mm^3^; *p* = 0.004). The concurrent enlargement at multiple levels suggests a globally dilated aortic root phenotype that is inherently resistant to achieving a complete seal, even with procedural optimization.

### 4.3. Pre-Procedural CT: From Conventional Sizing to Volumetric Learning

CT-based annular sizing has played a pivotal role in reducing PVL by enabling accurate prosthesis–annulus matching and revealing the extent and distribution of calcification [12]. However, current intra-procedural PVL assessment—relying on echocardiography, angiography, and hemodynamic indices—evaluates PVL only after it has occurred and remains limited by inter-observer variability and semi-quantitative grading [25,26,27,28]. More critically, no existing method provides reliable pre-procedural prediction of which patients will develop residual PVL despite optimization. Although the incidence of ≥moderate PVL has declined from over 20% with first-generation devices [29] to approximately 5% with contemporary valve designs [30], the prognostic impact of residual PVL remains substantial, underscoring the need for a predictive rather than reactive approach.

Our study extends the utility of pre-procedural CT beyond conventional annular sizing. While Model A demonstrated that manually derived CT parameters provide moderate predictive value (AUC 0.694), Model B’s 3D ResNet encoder—operating on the original CT volumes together with expert-segmented anatomical masks—achieved an AUC of 0.822, potentially capturing spatial patterns of root geometry and calcification distribution that are inherently lost in standard linear or area-based measurements.

### 4.4. Value of the Multi-Modal Deep Learning Approach

A recent systematic review of machine learning for TAVI outcome prediction [13] identified several models using tabular clinical and CT-derived features, with AUCs generally ranging from 0.65 to 0.80 for PVL-related outcomes. Our Model A (AUC 0.694) falls within this range, confirming the performance ceiling of conventional tabular approaches. Model B achieved a mean AUC of 0.822 with a specificity of 0.971, representing an absolute AUC improvement of 0.128 and exceeding the benchmark established by existing models, although these results require external validation given the modest sample size.

This performance gain may be attributable to three key design features. First, the two-stage training strategy decouples representation learning from outcome supervision: pretraining the 3D ResNet encoder on calcification volume—a continuous anatomical target mechanistically linked to PVL—provides an informative initialization that end-to-end training on only 35 events cannot achieve. Because calcification volume was also available to Model A as a scalar input, Model B’s advantage likely reflects its capacity to capture spatial and geometric patterns beyond what a single summary statistic encodes. Second, the use of expert-segmented masks as additional input channels enables the encoder to learn directly from structured spatial anatomy. Third, the cross-attention fusion mechanism dynamically weights anatomical regions in the context of each patient’s clinical profile, potentially capturing subtle morphological patterns—such as local curvature variations, calcification protrusion geometry, and asymmetric root deformation—that are difficult to quantify with manual measurements. Consistent with this hypothesis, gradient-based class activation mapping (Figure 5) demonstrated that Model B’s predictions were driven by spatially coherent activations across the ascending aorta, aortic annulus, and LVOT—anatomical levels that also showed significant enlargement in patients with residual ≥moderate PVL—supporting the biological plausibility of the model’s learned representations.

While the current study employed manual expert segmentation, this design establishes a pipeline that can be readily integrated with automated segmentation tools as they mature.

### 4.5. Clinical Implications

In our cohort, 9.0% of patients had residual ≥moderate PVL despite procedural optimization. While balloon post-dilatation and plug-based closure can reduce PVL severity [11,31,32,33], a proportion of patients remain with clinically significant residual regurgitation, underscoring the need for pre-procedural risk stratification rather than relying solely on intra-procedural rescue strategies.

Our findings support a personalized interventional approach. While the conventional model (Model A) provides a useful baseline risk estimate, Model B offers superior discrimination (AUC 0.822 vs. 0.694). Notably, Model B achieved a PPV of 0.860, indicating that the majority of patients flagged as high-risk did have residual PVL—a substantial improvement over Model A (PPV 0.488). For patients identified as high-risk by Model B, operators may consider alternative approaches upfront, such as selection of a different valve platform, intentional oversizing, pre-planned valve-in-valve, or preparation for plug-based closure.

### 4.6. Limitations

Several limitations should be acknowledged. First, this was a retrospective, single-center study with inherent selection bias; external validation in independent cohorts is required. Second, the modest number of outcome events warrants caution against overinterpretation. Performance metrics derived from small event cohorts, especially positive predictive value and specificity, are inherently statistically fragile and may fluctuate substantially with minor changes in the decision threshold or validation sample. External validation in larger, independent BAV-TAVI cohorts is essential to establish the true clinical utility and stability of the model. Third, the evolution of TAVI devices, techniques, and operator experience over the 8-year study period (2016–2024) may introduce temporal confounding. A further limitation is that the present study relies on expert manual segmentation of the aortic root and calcification, which is time-consuming and not directly suitable for routine clinical workflow; we regard this work as a preliminary step toward an automated pipeline, which is the focus of our ongoing research.

## 5. Conclusions

Among BAV patients undergoing TAVI with self-expanding valves, residual ≥moderate PVL—a complication independently linked to increased mortality—was associated with a distinct anatomical profile characterized by larger aortic root dimensions at all levels and greater calcification burden. The conventional prediction approach combining clinical variables with manually measured CT measurements achieved an AUC of 0.694, while the multi-modal deep learning model integrating segmentation-guided CT imaging and clinical variables via cross-attention achieved an AUC of 0.822 with a specificity of 0.971 in the current evaluation cohort, representing a substantial improvement. If validated externally, such a model could be incorporated into the pre-procedural CT analysis workflow to identify high-risk patients and support anatomy-driven procedural decision-making in BAV-TAVI.

## Figures and Tables

**Table 1:** Baseline Clinical, Anatomical, Echocardiographic, and Procedural Characteristics.

Variable	All (N = 402)	<Moderate PVL (*n* = 366)	≥Moderate PVL (*n* = 36)	*p* Value
Clinical characteristics				
Male, *n* (%)	228 (56.7%)	201 (54.9%)	27 (75.0%)	0.032
Age, yrs	72 (67–77)	72 (67–76)	74 (70–78)	0.031
BMI, kg/m^2^	22.6 (20.4–24.9)	22.7 (20.5–25.0)	21.7 (19.6–24.1)	0.201
STS score, %	2.75 (1.83–4.70)	2.71 (1.78–4.68)	3.31 (2.13–4.74)	0.193
Hypertension, *n* (%)	123 (30.6%)	114 (31.1%)	9 (25.0%)	0.566
Diabetes, *n* (%)	72 (17.9%)	70 (19.1%)	2 (5.6%)	0.041
COPD, *n* (%)	62 (15.4%)	56 (15.3%)	6 (16.7%)	1.000
Coronary artery disease, *n* (%)	95 (23.6%)	89 (24.3%)	6 (16.7%)	0.409
Chronic kidney disease, *n* (%)	21 (5.2%)	19 (5.2%)	2 (5.6%)	1.000
Atrial fibrillation, *n* (%)	35 (8.7%)	30 (8.2%)	5 (13.9%)	0.397
Peripheral vascular disease, *n* (%)	74 (18.4%)	70 (19.1%)	4 (11.1%)	0.365
Prior stroke/TIA, *n* (%)	43 (10.7%)	40 (10.9%)	3 (8.3%)	0.783
CT-derived aortic root anatomy				
AV calcification volume, mm^3^	578.0 (296.0–933.6)	559.2 (292.6–914.6)	887.6 (566.1–1233.5)	0.004
Aortic annular angle, °	53.9 (48.0–59.9)	53.9 (48.1–59.6)	55.7 (47.6–63.4)	0.360
Annular perimeter, mm	78.6 (72.1–84.9)	78.0 (72.0–84.3)	82.7 (76.4–92.2)	0.003
Annular area, mm^2^	475.2 (401.5–551.2)	466.7 (400.6–548.6)	524.6 (437.2–643.6)	0.010
SoV perimeter, mm	108.5 (100.6–118.5)	107.5 (100.2–118.0)	115.2 (108.1–129.2)	<0.001
STJ diameter, mm	30.8 (28.4–33.9)	30.5 (28.1–33.5)	34.8 (33.0–38.2)	<0.001
LCA ostium height, mm	14.1 (12.3–16.5)	14.1 (12.3–16.5)	15.6 (13.2–17.1)	0.076
RCA ostium height, mm	15.0 (12.9–17.5)	15.0 (12.9–17.4)	15.1 (13.5–18.0)	0.541
Max ascending Ao diameter, mm	42 (39–46)	42 (38–45)	45 (42–48)	<0.001
LVOT perimeter, mm	83.6 (75.0–92.9)	83.3 (74.8–92.2)	91.8 (77.4–101.4)	0.007
Echocardiographic parameters				
AR (≥moderate), *n* (%)	52 (12.9%)	45 (12.3%)	7 (19.4%)	0.337
Mean AV gradient, mmHg	59 (44–74)	59 (44–73)	56 (46–75)	0.949
Peak AV velocity, m/s	4.9 (4.3–5.5)	4.9 (4.3–5.5)	4.8 (4.4–5.5)	0.650
LVEF, %	60 (43–67)	60 (44–67)	55 (38–62)	0.057
LVEDD, mm	50 (45–56)	49 (45–55)	55 (50–61)	0.002
IVS, mm	14 (12–15)	14 (12–15)	14 (12–15)	0.461
Procedural characteristics				
New-generation THV, *n* (%)	155 (38.6%)	143 (39.1%)	12 (33.3%)	0.620
THV size <26 mm, *n* (%)	136 (33.8%)	127 (34.7%)	9 (25.0%)	0.323
Transfemoral access, *n* (%)	398 (99.0%)	363 (99.2%)	35 (97.2%)	0.314
Pre-dilatation, *n* (%)	381 (94.8%)	349 (95.4%)	32 (88.9%)	0.107
Post-dilatation balloon size, mm	20 (20–22)	20 (20–22)	22 (20–24)	0.008

Values are median (Q1–Q3) or *n* (%). ≥Moderate PVL defined as residual ≥moderate paravalvular leak after completion of intra-procedural optimization. AV, aortic valve; BMI, body mass index; COPD, chronic obstructive pulmonary disease; IVS, interventricular septum thickness; LCA, left coronary artery; LVEDD, left ventricular end-diastolic diameter; LVEF, left ventricular ejection fraction; LVOT, left ventricular outflow tract; PVL, paravalvular leak; SoV, sinus of Valsalva; STS, Society of Thoracic Surgeons; STJ, sinotubular junction; THV, transcatheter heart valve; TIA, transient ischemic attack.

**Table 2:** Model Performance.

	Model A (Conventional)		Model B (Multi-Modal DL)	
	Mean ± SD	95% CI	Mean ± SD	95% CI
AUC	0.694 ± 0.050	0.596–0.792	0.822 ± 0.114	0.680–0.964
Accuracy	0.781 ± 0.136	0.514–1.000	0.881 ± 0.038	0.834–0.928
Sensitivity	0.657 ± 0.217	0.233–1.000	0.429 ± 0.202	0.178–0.680
Specificity	0.806 ± 0.200	0.413–1.000	0.971 ± 0.049	0.910–1.000
PPV	0.488 ± 0.194	0.108–0.869	0.860 ± 0.219	0.588–1.000
NPV	0.930 ± 0.043	0.846–1.000	0.896 ± 0.030	0.859–0.933

Model A: clinical and procedural variables + manually measured CT variables, Random Forest classifier, 5-fold stratified cross-validation. Model B: multi-modal deep learning (3D ResNet + MLP + cross-attention), with 5-fold stratified cross-validation. AUC, area under the receiver operating characteristic curve; CT, computed tomography; DL, deep learning; NPV, negative predictive value; MLP, multilayer perceptron; PPV, positive predictive value.

## Data Availability

The data presented in this study are available from the corresponding author upon reasonable request. The deep learning code is available from the corresponding author upon reasonable request. Restrictions apply to the availability of CT imaging data due to patient privacy considerations.

## Supplementary Materials

The following supporting information can be downloaded at: https://www.mdpi.com/article/10.3390/biomedicines14040946/s1, Table S1: Cross-validated performance metrics of five machine learning classifiers; Table S2: Detailed performance metrics for Model A and B across individual folds of the 5-fold cross-validation.

## Abbreviations

The following abbreviations are used in this manuscript:

AUC	Area under the receiver operating characteristic curve
BAV	Bicuspid aortic valve
BCE	Binary cross-entropy
CT	Computed tomography
DCA	Decision curve analysis
GBM	Gradient boosting machine
Grad-CAM	Gradient-weighted Class Activation Mapping
LR	Logistic regression
LVEDD	Left ventricular end-diastolic diameter
LVEF	Left ventricular ejection fraction
LVOT	Left ventricular outflow tract
MLP	Multilayer perceptron
NPV	Negative predictive value
PPV	Positive predictive value
PVL	Paravalvular leak
ResNet	Residual network
RF	Random Forest
ROC	Receiver operating characteristic
SoV	Sinus of Valsalva
STJ	Sinotubular junction
STS	Society of Thoracic Surgeons
TAVI	Transcatheter aortic valve implantation
THV	Transcatheter heart valve
