# Supplementary material for: Neural Network-Based Prediction of Residual Paravalvular Leak in Bicuspid Aortic Valve TAVI Using CT-Derived Anatomical Features
Source: Biomedicines. 2026 Apr 21;14(4):946. doi: 10.3390/biomedicines14040946 (PMC13113058; doi:10.3390/biomedicines14040946)
Supplement: Supplementary file 1 [file biomedicines-14-00946-s001.zip › biomedicines-4233795-supplementary.pdf]

# Supplementary Material

- **Supplementary Table S1.** Cross-validated performance metrics of five machine learning classifiers.
- 
- **Supplementary Table S2.** Detailed performance metrics for Model A and B across individual folds of the 5-fold cross-validation.

**Supplementary Table S1.** Cross-validated performance metrics of five machine learning classifiers.

| | **AUC** | **Accuracy** | **Sensitivity** | **Specificity** | **PPV** | **NPV** |
|---|---|---|---|---|---|---|
| LR (L2) | 0.629 ± 0.129 | 0.567 ± 0.137 | 0.743 ± 0.212 | 0.531 ± 0.135 | 0.250 ± 0.083 | 0.903 ± 0.101 |
| LR (L1) | 0.627 ± 0.128 | 0.686 ± 0.170 | 0.657 ± 0.260 | 0.691 ± 0.207 | 0.345 ± 0.135 | 0.910 ± 0.081 |
| MLP | 0.587 ± 0.052 | 0.581 ± 0.174 | 0.771 ± 0.217 | 0.543 ± 0.251 | 0.280 ± 0.077 | 0.941 ± 0.054 |
| **RF** | 0.694 ± 0.050 | 0.781 ± 0.136 | 0.657 ± 0.217 | 0.806 ± 0.200 | 0.488 ± 0.194 | 0.930 ± 0.043 |
| GBM | 0.692 ± 0.084 | 0.676 ± 0.174 | 0.771 ± 0.163 | 0.657 ± 0.231 | 0.398 ± 0.238 | 0.941 ± 0.041 |

All metrics are presented as mean ± standard deviation across 5 stratified folds. Random Forest (RF) was selected as the final classifier for Model A because it achieved the highest discriminative performance (AUC) and the highest specificity, which is clinically critical for minimizing false-positive predictions in this low-event cohort.

AUC, area under the receiver operating characteristic curve; GBM, gradient boosting machine; LR (L1), L1-regularized logistic regression (LASSO); LR (L2), L2-regularized logistic regression (Ridge); MLP, multilayer perceptron; NPV, negative predictive value; PPV, positive predictive value; PVL, paravalvular leak; RF, Random Forest.

**Supplementary Table S2.** Detailed performance metrics for Model A and B across individual folds of the 5-fold cross-validation.

| | **Fold 1** | **Fold 2** | **Fold 3** | **Fold 4** | **Fold 5** | **Mean ± SD** |
|---|---|---|---|---|---|---|
| **Model A** | | | | | | |
| AUC | 0.759 | 0.735 | 0.647 | 0.669 | 0.659 | 0.694±0.050 |
| Accuracy | 0.905 | 0.833 | 0.810 | 0.810 | 0.548 | 0.781±0.136 |
| Sensitivity | 0.571 | 0.714 | 0.429 | 0.571 | 1.000 | 0.657±0.217 |
| Specificity | 0.971 | 0.857 | 0.886 | 0.857 | 0.457 | 0.806±0.200 |
| PPV | 0.800 | 0.500 | 0.429 | 0.444 | 0.269 | 0.488±0.194 |
| NPV | 0.919 | 0.938 | 0.886 | 0.909 | 1.000 | 0.930±0.043 |
| **Model B** | | | | | | |
| AUC | 0.931 | 0.690 | 0.951 | 0.751 | 0.788 | 0.822±0.114 |
| Accuracy | 0.833 | 0.857 | 0.881 | 0.905 | 0.929 | 0.881±0.038 |
| Sensitivity | 0.571 | 0.143 | 0.286 | 0.571 | 0.571 | 0.429±0.202 |
| Specificity | 0.886 | 1.000 | 1.000 | 0.971 | 1.000 | 0.971±0.049 |
| PPV | 0.500 | 1.000 | 1.000 | 0.800 | 1.000 | 0.860±0.219 |
| NPV | 0.912 | 0.854 | 0.875 | 0.919 | 0.921 | 0.896±0.030 |

AUC, area under the receiver operating characteristic curve; NPV, negative predictive value; PPV, positive predictive value; SD, standard deviation.
